# Perception and attitude of specialist nurses on “Internet + Nursing Service”: a mixed-methods study with qualitative exploration from patients and families

**DOI:** 10.3389/fpubh.2026.1779884

**Published:** 2026-06-29

**Authors:** Mengfan Ma, Daishan Li, Wangao Guan, Yamei Zuo, Zilin Ma, Yi Dai

**Affiliations:** 1 Northeast Yunnan Central Hospital, Zhaotong, China; 2 The First People's Hospital of Yunnan Province/The Affiliated Hospital of Kunming University of Science and Technology, Kunming, China; 3 Kunming Municipal Hospital of Traditional Chinese Medicine, Kunming, China

**Keywords:** attitude, Internet + Nursing Service, mixed-methods research, perception, specialist nurses

## Abstract

**Background:**

The implementation of “Internet + Nursing Service” in economically underdeveloped and culturally diverse regions, such as Yunnan Province, China, presents particular challenges, although it has emerged as a crucial solution to address global aging concerns.

**Aim:**

In addition to qualitatively examining the viewpoints of patients and families as service recipients, this study aimed to understand how specialized nurses perceived and felt about the “Internet + Nursing Service.”

**Methods:**

From 1 January to 30 June 2024, a convergent mixed-methods study was carried out. In Yunnan Province, China, a total of 1,200 specialist nurses participated in a cross-sectional survey. Twelve participants—five patients and seven family caregivers—who had used an online nursing service participated in semi-structured interviews at the same time. Multiple linear regression, univariate analysis, and descriptive statistics were used to analyze quantitative data. Thematic analysis was used to examine qualitative data. The quantitative results were explained, contextualized, and elaborated upon using the qualitative themes.

**Results:**

The attitude of specialist nurses toward “Internet + Nursing Service” was good (mean 3.90 ± 0.69), but their perception was only moderate (mean 3.31 ± 0.64). Nurses' perception scores were substantially correlated with employment position, educational level, institutional implementation, and individual participation (all *p* < 0.05), according to the regression analysis. Convenience was cited as a major advantage in qualitative interviews with patients and family caregivers, which supported this optimistic outlook. The thematic analysis, however, uncovered a complex “awareness-sophistication gap” marked by a lack of comprehension of the service's specific requirements and a superficial familiarity with it. The analysis found that economic constraints and safety concerns were important moderators of acceptance. The comprehensive study shows that the main challenges to service scale-up are not attitudinal resistance but rather informational and structural limitations.

**Conclusion:**

Despite the positive perceptions of “Internet + Nursing Service” held by both service recipients and specialist nurses, there remains a substantial knowledge gap. Along with unresolved safety concerns and budgetary barriers, this gap is a significant lever for intervention. Hospital administrators and policymakers should prioritize targeted awareness campaigns that elucidate the unique nature of the service, alongside structural improvements intended to enhance safety protocols and expand insurance coverage.

## Introduction

Population aging has become a significant global public health concern as a result of the rapid growth of the population (1). According to the most recent national census conducted in 2020, 14% of Chinese people were 65 years of age or older, indicating that China's population is rapidly aging (2). Approximately 210 million people, or 80.1% of China's older adult population, suffer from chronic conditions that put them at serious risk of impairment (3). In addition to putting significant strain on pension systems, China's social security, health, and medical systems are also seriously threatened by the country's aging population, which has an impact on both population quality and the development of human capital (4).

China officially began the “Pilot Work Programme on Carrying Out ‘Internet + Nursing Service”' in response to these difficulties (5). The phrase “Internet + Nursing Service” refers to nursing care provided by facility-registered nurses using an “online application and offline service” paradigm to discharged patients or specific populations with mobility and illness-related demands (5). In a number of China's economically developed coastal cities, this service has advanced significantly and proven beneficial (6–8). Home-based nursing services backed by digital platforms have been popular worldwide as an affordable way to reduce hospital readmission rates and increase patient satisfaction (9, 10). Research from high-income nations, such as the United States (11) and Australia (12), has demonstrated that these services can reduce caregiver burden and improve care continuity.

However, the majority of the evidence currently available comes from environments with strong digital infrastructure, well-established insurance systems, and high levels of health literacy. This raises concerns about the model's applicability in middle-income or resource-constrained settings, especially those marked by lower economic development, ethnic diversity, and geographic dispersion.

There are still several significant gaps in the growing corpus of research on Internet + Nursing Service. First, Yunnan Province, an inland, multiethnic, and economically underdeveloped region of China, has not yet had its attitudes and views toward this service thoroughly investigated. This disparity is important because regional differences in institutional capacity, health literacy, and infrastructure may create particular obstacles not encountered in research conducted in coastal towns with abundant resources. Second, rather than combining the two viewpoints to fully comprehend service acceptance, previous research has mostly focused on provider perceptions and recipient experiences separately. Third, because much previous research has relied solely on quantitative methods, which can uncover associations but cannot explain their underlying causes, the mechanisms linking institutional issues to individual views remain poorly understood.

Additionally, because Yunnan Province's Internet + Nursing Service is still in its early stage of development and service utilization remains limited, a strictly quantitative study would not have sufficient statistical power. On the other hand, from the perspective of specialized nurses, qualitative research can offer more targeted recommendations for improvement and a deeper understanding of patients' specific needs, expectations, and satisfaction as service recipients whose experiences are likely to be complex and varied.

To make the most of the limited sample resources, achieve a more comprehensive research perspective, and gain deeper insights, this study employs a mixed-methods approach. This approach adds new knowledge from an international perspective in three distinct ways: (1) it challenges the notion that findings from coastal cities are universally applicable to all Chinese or Asian contexts by providing empirical evidence from Yunnan Province, China; (2) it employs a convergent mixed-methods design to integrate provider perceptions (quantitative) with recipient experiences (qualitative), providing a more comprehensive understanding of service acceptance barriers than either method alone could achieve; and (3) it identifies region-specific leverage points for policy intervention that may be relevant to similar contexts worldwide, such as rural Southeast Asia, parts of Latin America, and underserved areas in high-income countries where workforce shortages and digital divides remain.

The following research questions were specifically addressed in this study: (1) How do nurses in Yunnan Province feel about Internet + Nursing Services? (2) What factors are associated with nurses' perception and attitude scores, such as personal involvement, institutional support, and demographics? (3) How are the quantitative results explained, contextualized, or expanded upon by the experiences and concerns of patients and families who have utilized Internet + Nursing Service? No *a priori* hypotheses were developed for the qualitative research topic because the qualitative component was exploratory in nature. We hypothesized the following quantitative research questions, including (H1) specialist nurses would report strongly positive attitudes and moderately positive perceptions of Internet + Nursing Service, in line with previous studies; and (H2) institutional implementation and individual participation would be significant positive predictors of perception scores, after controlling for demographic variables.

## Methods

### Design and sample

This study employed a convergent mixed-methods strategy. The design included qualitative interview data from patients and their family caregivers, along with quantitative survey data from specialized nurses. Since both strands were deemed necessary to answer the study questions, they were gathered simultaneously between 1 January and 30 June 2024, with equal priority (QUAN + QUAL) given to each strand. Integration was intended to take place at the interpretation stage; neither the quantitative nor the qualitative strand was given priority over the other. This equal-priority convergent design was chosen because neither method could adequately address the research questions on its own: while qualitative data could explain why certain patterns exist and how they appear in lived experience, quantitative data could identify levels and predictors of perceptions and attitudes.

The semi-structured interview guide for patients and family caregivers was refined using preliminary descriptive statistics from the 1,200 nurse surveys, even though the two strands were gathered simultaneously. In particular, the initial finding that nurses' perception scores were only moderate (mean 3.31 ± 0.64) despite positive attitudes (mean 3.90 ± 0.69) led to the addition of probes that examined patients' and families' understanding of the specialized qualification requirements of Internet + Nursing Service providers. To ensure heterogeneity in experiences, patients and families from both secondary and tertiary hospitals were recruited in response to the initial observation that perception scores differed by hospital level.

The weaving technique was used to accomplish integration at the interpretation phase. To facilitate side-by-side comparison, a combined display table (Table 1) is provided in the Results section. For instance, as described in the Results section, the significant statistical relationship between institutional implementation and nurses' perception scores was interpreted using qualitative data on safety and cost concerns.

**Table 1:** Joint display of integrated findings: quantitative patterns, qualitative themes, and interpretive integration.

Integrated theme	Quantitative finding	Qualitative theme/exemplary quote	Interpretive integration
Theme 1: moderate awareness with a nuanced understanding gap	Mean perception score = 3.31 ± 0.64 (moderate, slightly positive on 5-point scale)	“I don't think there should be any special requirements for home-based care; as long as you have a nursing license, it should be sufficient.” (B11, Family caregiver)	The “awareness-sophistication gap”—superficial familiarity coexisting with a fundamental misunderstanding of specialized qualification requirements—is reflected in the modest quantitative score rather than just restricted awareness.
Theme 2: positive attitudes as conditional acceptance	The univariate analysis revealed that female nurses and those working in higher-level hospitals had significantly greater positive attitudes (p < 0.05); the mean attitude score was 3.90 ± 0.69 (positive).	Convenience driver: “We no longer need to go to the hospital in person and wait in line; this saves us a lot of time and energy.” (B8, Patient)	Sincere but conditional optimism is tempered by financial barriers and safety concerns, which prevent good sentiments from being translated into real service uptake. This explains why even positive-minded nurses could experience patient resistance or sense minimal demand.
		Safety moderator: “Receiving treatment at home is not the same as in the hospital; without resuscitation equipment, it could be dangerous if something happens.” (B2, Patient)	
		Cost moderator: “The service is good, but the price… we have to think carefully every time we place an order. We hope it can be covered by medical insurance.” (B4, Family caregiver)	
Theme 3: structural barriers to scale-up	Perception scores were significantly predicted by institutional implementation (β = 0.35, *p* < 0.001) and individual participation (β = 0.10, *p* = 0.003) (multiple linear regression).	Fee system: “We hope the costs will be covered by medical insurance. Otherwise, for families like ours, it's a luxury we can only afford occasionally.” (B4, Family caregiver)	The statistical predictor “institutional implementation” is more than just a statistical artifact; it reflects actual structural elements that significantly influence provider attitudes and service uptake, such as safety procedures, fee transparency, and digital accessibility.
		Safety protocols: “If something goes wrong at home, who is responsible? In the hospital, there are protocols and emergency teams.” (B2, Patient)	
		Digital literacy: “My mother is 82. She can't use the app at all. I have to help her every time. Some of her friends don't have children nearby—how would they even access this service?” (B7, Family caregiver)	

After obtaining informed consent, specialist nurses who met the inclusion criteria were recruited. The inclusion criteria included: (1) being within the active registration period and possessing a valid Chinese nursing practice certification; (2) having completed rigorous training for specialist nurses and passed the relevant examinations, thereby obtaining certification in the relevant specialty; and (3) being capable of independently providing the “Internet + Nursing Service” on their own after receiving instruction. The exclusion criteria included (1) inability to complete the questionnaire for any reason during the study period; and (2) refusal to participate in the study.

### Quantitative data collection

Since it was impractical to contact the target population, which was dispersed throughout Yunnan Province, convenience sampling was used to recruit specialized nurses. This survey was conducted using an online self-administered questionnaire. The survey was developed specifically for this study. A review of national policies, the local context, and relevant literature informed the initial draft, which included general information and perception/attitude measures.

Five experts participated in a Delphi review to evaluate content validity (authority coefficient = 0.933). The item-level indices (I-CVI) ranged from 0.8 to 1.0, whereas the scale-level Content Validity Index (S-CVI) was 1.0. Thirty specialist nurses who were not included in the main study participated in a pilot test. The perception subscale (15 items) and attitude subscale (10 items) showed Cronbach's α values of 0.89 and 0.91, respectively, suggesting strong internal consistency. The scale's overall Cronbach's α was 0.925.

A 5-point Likert scale with the following anchors was used for all perception and attitude items: 1 = strongly disagree, 2 = disagree, 3 = neutral, 4 = agree, and 5 = strongly agree. Knowledge of Internet + Nursing Service policies (such as “I am familiar with the national pilot work program for Internet + Nursing Service”), comprehension of service procedures (such as “I know the steps for online order placement and offline service delivery”), and knowledge of qualification requirements (such as “I am aware that only certified specialist nurses are eligible to provide Internet + Nursing Service”) were all evaluated using the perception subscale. Affective and behavioral inclinations were measured using the attitude subscale (such as “I believe Internet + Nursing Service is a valuable complement to hospital care”; and “I would recommend Internet + Nursing Service to eligible patients”). The mean item score (range 1–5) was used to calculate the scale scores for perception and attitude, with higher scores denoting a more positive perception or attitude. A mean score of 3.0 was deemed neutral, scores above 3.5 showed a positive tendency, and scores below 2.5 indicated a negative tendency, according to the standard interpretation for Likert-type scales in healthcare research.

A secure connection was created using Sojump (Changsha Ranxing Information Technology Co., Ltd., China), a reputable online survey platform frequently used in Chinese health research, to distribute the final questionnaire. The nursing directors of 47 hospitals in Yunnan Province received comprehensive research explanations and were requested to cooperate with the researchers. The directors then sent the secure link to the facilities' eligible specialist nurses. Because the target demographic was geographically distributed throughout Yunnan Province's hilly terrain and face-to-face recruiting was logistically impractical, face-to-face distribution was not performed. Only the secure link could be used to access the questionnaire. Before accessing the questionnaire, each participant had to provide digital informed consent. To avoid duplicate responses, IP address restrictions were set to allow only one submission per address, and participants were required to answer all questions. The final analysis did not include any invalid questionnaires, such as those with straight-line answers, logical flaws, or completion times shorter than 3 min.

### Quantitative data analysis

The data were examined using SPSS 26.0 (IBM, USA). Categorical variables were described using frequency (percentage), whereas continuous variables were described using mean (standard deviation). Differences in perception and attitude scores were examined using the Welch's *t*-test and chi-square test. Multiple linear regression analysis was performed to identify the components influencing perception ratings. Prior to regression, assumptions were evaluated. Multicollinearity was assessed using Variance Inflation Factors (VIFs); all values below 2.0 indicated no significant issue. A graphical evaluation of the residuals' homoscedasticity and normality revealed that they were both acceptable. The significance threshold was set at α = 0.05. The following categories were dummy-coded prior to regression analysis: educational level (such as, 1 = junior college or lower, 2 = bachelor's degree, and 3 = master's degree or higher), job position (such as, 1 = nurse, 2 = head nurse, and 3 = director of nursing), institutional implementation (1 = yes or 2 = no), and individual participation (1 = yes or 2 = no). The Harman's single-factor test was conducted to assess common method bias (CMB). The first component accounted for 28.7% of the overall variance, below the suggested threshold of 50%. An unrotated exploratory factor analysis on all perceptual and attitude measures suggested that CMB was not a major concern in this study (13).

### Qualitative data collection

To ensure a broad range of experiences, patients who had used Internet + Nursing Service and their family caregivers from three hospitals in Yunnan Province—two tertiary and one secondary—were specifically chosen for semi-structured interviews.

Patients were included when they: (1) had received Internet + Nursing Service at least once; (2) could use the Internet + Nursing Service for ordering applications or had a family member who could; and (3) had normal cognitive function and the capacity for verbal or written communication. If a family caregiver assisted a patient use Internet + Nursing Service, they were also included. Both groups were excluded if they were unwilling to participate or were unable to complete the interview because of a cognitive or physical impairment.

Three steps were taken in the development of the semi-structured interview guide. To identify common domains investigated in studies of patients' and families' experiences with home-based and online nursing services, this study first reviewed the literature. The initial domain structure was informed by pertinent earlier research (14, 15). Second, an initial pool of 12 open-ended questions covering four areas was created by the study team (comprising two clinical nursing supervisors), including (1) general perceptions of Internet + Nursing Service; (2) perceived benefits and downsides; (3) concerns and barriers, and (4) suggestions for enhancement. Four individuals who had utilized the Internet + Nursing Service—two patients and two family caregivers—were given a pre-test to evaluate the acceptability, clarity, and fluency of the questions. These participants were not included in the main study. In response to pilot feedback, two questions were reworded to avoid using leading language (such as, “How has Internet + Nursing Service benefited you?” was changed to “In what ways, if any, has Internet + Nursing Service affected your or your family member's care situation?”), and one question about “willingness to pay” was moved to the end of the guide to prevent priming earlier responses.

Prior to each interview, the interviewer—a qualified qualitative researcher with a background in nursing—spends about 10 min introducing herself, outlining the goal of the study, guaranteeing confidentiality, and engaging in off-topic small talk (such as discussing the participant's family or daily routine). Second, technical jargon (such as “specialist nurse certification”) was either avoided or described in plain language, and all interviews were performed in the participant's chosen language (Mandarin or, when necessary, with support from a local dialect speaker). Third, the interviewer halted, reassured the participant, and offered them the option to skip the subject or take a break when they showed signs of doubt or emotional distress (such as when discussing financial concerns). Fourth, the interviewer employed a responsive listening technique for family caregivers of patients over 65, asking questions such as “So what I hear you saying is that safety is your biggest concern, is that correct?” to ensure that participants understood. Due to communication breakdowns, no interviews were necessary for an early termination.

After obtaining informed consent, eligible respondents who had first been contacted through phone or email with a brief explanation of the study's objectives were interviewed in person for 30–40 min. With participants' consent, all interviews were audio recorded. Within 48 h following each interview, the audio recording was verbatim transcribed. Each transcript was provided to the appropriate participant through encrypted email for member verification to ensure accuracy. Participants were requested to check the transcript for correctness, provide clarification on any unclear statements, and make any necessary additions or deletions. No participants asked for significant revisions to their views; however, three participants (25%) returned with minor corrections (mostly grammatical or wording clarifications). All subsequent analyses were conducted using corrected transcripts. According to Saunders' criterion, saturation was determined after ten interviews, and two additional interviews were performed to validate it (16).

### Qualitative data analysis

The interviews were transcribed word-for-word. The transcripts were analyzed using NVivo 11.0. To establish initial categories, two analysts independently performed open coding. The use of axial coding to create connections between categories led to the identification of primary themes. Selective coding was used to blend these themes into a coherent narrative that addressed the primary research questions.

There were three members in the research team. With 2 years of formal training in qualitative research methods (including graduate-level courses in qualitative methodology, NVivo training, thematic analysis, and reflexivity practice), the lead researcher (first author) is a female PhD candidate in nursing with 10 years of clinical experience as a registered nurse. The second author is a female nursing professor with 15 years of expertise in qualitative supervision and mixed-methods research. To prevent bias, the third author, a male specialty nurse with 3 years of expertise in home care services, helped with participant recruiting and offered clinical context but did not participate in data coding or analysis.

Two analysts (the primary researcher and a research assistant with a master's degree in nursing and training in qualitative methodologies) separately performed open coding to determine preliminary categories. A formal evaluation of inter-rater reliability was conducted. Using an initial codebook created from open coding of the first transcript, the two analysts separately coded the same three transcripts (25% of the sample, chosen to represent patients, family caregivers, and diverse service experiences). Each code category's Cohen's kappa coefficient was determined; the average kappa across codes was 0.84 (range: 0.76–0.91), showing significant to excellent agreement. Code definitions were discussed and improved to settle disagreements (mostly over border judgments between “safety concerns” and “responsibility concerns”). The lead researcher independently coded the remaining nine transcripts, while the second author reviewed an additional two transcripts (16.7%) to ensure continued consistency. The use of axial coding to create connections between categories led to the identification of main themes. A coherent story that addressed the primary study questions was created by combining these themes through selective coding.

### Integration of quantitative and qualitative strands

Strategy 1: Weaving during the interpretation phase. The study team conducted several integration sessions after the quantitative and qualitative data were analyzed concurrently but independently. We methodically matched qualitative themes (such as safety concerns, cost obstacles, and digital literacy) with quantitative data (such as mean perception score = 3.31; β for institutional implementation = 0.35) during these sessions. The study questioned directly: “How do the qualitative themes help explain or contextualize this quantitative pattern?” The three integrated themes shown in the results were produced through this iterative process.

Strategy 2: Joint display represents the second strategy. For each of the three integrated themes, a joint display table (Table 1) was created to graphically compare numeric results, qualitative themes/exemplar quotes, and interpretative integration. Readers can directly evaluate the integration's coherence and explanatory usefulness thanks to this side-by-side presentation.

Strategy 3: Integration of narrative in discussion. The discussion explicitly revisits each integrated theme after the Results section, offering conclusions and contrasting results with previous research. This three-level integration (Results table + Results narrative + Discussion) guarantees that integration is integrated throughout the article rather than just in one place.

The GRAMMS (Good Reporting of A Mixed Methods Study) checklist is followed in the reporting of this mixed-methods study (17). There are six items on the checklist. Each item's treatment in this study is summed up in Table 2.

**Table 2:** GRAMMS checklist compliance.

GRAMMS item	Where addressed in this manuscript
1. Justification for using mixed methods	Introduction (paragraph 4–5)
2. Design description (convergent, parallel, or sequential)	Methods—Design and sample
3. Description of integration process (timing, priority, weaving approach)	Methods—Design and sample; Methods—Integration of quantitative and qualitative strands
4. Treatment of divergences/contradictions between strands	Discussion—Synthesis of mixed-methods insights (note: no major contradictions were identified)
5. Discussion of how integration influences findings	Results—Integrated Themes (Themes 1–3); Discussion—Synthesis
6. Limitations of mixed methods (e.g., integration challenges)	Discussion—Strengths and limitations

### Ethical considerations

In this study, the Declaration of Helsinki was adhered to. All data in this publication were collected as part of a larger, previously approved research study for a master's thesis. The full research protocol was approved by the Ethics Review Committee of the First People's Hospital of Yunnan Province (Approval number: KHLL2023-KY013). Each participant provided written informed consent that explicitly stated that their anonymized data could be used in future academic publications. The IRB approved the parent project on 29 January 2023. Respondents were informed that participation in the survey or interview was completely voluntary and that they could withdraw at any time, even after the study had begun.

## Results

### Participant characteristics

A total of 1,200 surveys were obtained from a potential population of 1,292, yielding an estimated response rate of 92.9%. Table 3 displays the descriptive statistics of the sample. Of the participants, 94.8% were women, with an average age of 35.83 ± 6.50 years and mean work experience of 13.25 ± 7.76 years. In terms of professional status, 60.2% held the title of supervising nurse and 91.9% had a bachelor's degree (Table 3). Twelve individuals participated in the semi-structured interviews. The sample included five patient participants (41.7%) and seven family caregiver participants (58.3%). Among the five patient participants, three (60.0%) were women with a mean age of 52.4 ± 12.30 years. Similarly, among the seven family caregiver participants, four (57.1%) were women with a mean age of 33.6 ± 9.80 years.

**Table 3:** Specialist nurses' perception to “Internet+Nursing Service” by different demographic characteristics (*n* = 1,200).

Characteristic	Results, *n* (%)	M ±SD	*t* or *F*	*p*
Gender			−1.56	0.119
Man	62 (5.2)	3.19 ± 0.74		
Woman	1,138 (94.8)	3.32 ± 0.63		
Age (years)			1.14	0.191
20–29	183 (15.3)	3.30 ± 0.59		
30–39	703 (58.6)	3.27 ± 0.64		
40–49	276 (23.0)	3.38 ± 0.66		
50–59	38 (3.2)	3.50 ± 0.61		
Working experience			1.18	0.141
≤ 4	51 (4.3)	3.15 ± 0.52		
5–10	241 (20.1)	3.28 ± 0.63		
10–20	658 (54.8)	3.28 ± 0.63		
>20	250 (20.8)	3.46 ± 0.67		
Professional title			1.25	0.069
Nurse	41 (3.4)	3.44 ± 0.64		
Senior nurse	335 (27.9)	3.32 ± 0.63		
Supervisor nurse	722 (60.2)	3.28 ± 0.63		
Co-chief nurse	97 (8.1)	3.45 ± 0.70		
Chief nurse	5 (0.4)	3.16 ± 0.52		
Job position			1.42	0.009
Nurse	988 (82.3)	3.28 ± 0.62		
Head nurse	211 (17.6)	3.44 ± 0.69		
Director of nursing	1 (0.1)	3.52		
Educational level			1.30	0.040
Junior college or lower	72 (6.0)	3.42 ± 0.70		
Bachelor's degree	1,103 (91.9)	3.30 ± 0.63		
Master's degree or higher	25 (2.1)	3.52 ± 0.61		
hospital type			−1.38	0.167
General hospital	1,058 (88.2)	3.30 ± 0.63		
Other specialist hospitals	142 (11.8)	3.38 ± 0.71		
Hospital level			1.21	0.102
Tertiary hospital	964 (80.3)	3.02 ± 0.63		
Secondary hospital	231 (19.3)	3.28 ± 0.69		
Primary hospital	5 (0.4)	3.32 ± 0.62		
Hospital nature			0.81	0.417
Public hospital	1,177 (98.1)	3.31 ± 0.64		
Private hospital	23 (1.9)	3.20 ± 0.63		
Authorized strength			0.46	0.644
Yes	323 (26.9)	3.32 ± 0.66		
No	877 (73.1)	3.30 ± 0.63		
The institution carrying out “Internet + Nursing Service”			0.12	< 0.001
Yes	482 (40.2)	3.56 ± 0.60		
No	718 (59.8)	3.14 ± 0.60		
Specialist nurse participated in”Internet+Nursing Service”			0.10	< 0.001
Yes	121 (10.1)	3.92 ± 0.65		
No	1,079 (89.9)	3.24 ± 0.60		

M, $\frac{1}{4}$ mean; SD, $\frac{1}{4}$ standard deviation.

### Research findings

On a 5-point Likert scale (1 = strongly disagree, 5 = strongly agree), specialty nurses' average perception score is 3.31 ± 0.64. A mean score of 3.0 is regarded as neutral, scores above 3.5 indicate positive leaning, and scores below 2.5 indicate negative leaning, according to the standard interpretation for the scales. As a result, the observed score points depicted a moderate, somewhat favorable perception. An overall good attitude is indicated by the average attitude score of 3.90 ± 0.69.

This study presents the details of each of the three integrated themes below, following the joint display in Table 1. The quantitative result, qualitative elaboration (with representative quotes), and interpretive integration—which shows how the qualitative data reframe, contextualize, or extend the quantitative pattern—are presented for individual subjects.

### Integrated theme 1: Moderate awareness with a nuanced understanding gap

Quantitative finding: A moderate level of awareness was indicated by the specialized nurses, with mean perception scores of 3.31 ± 0.64.

Qualitative elaboration: According to patient and family interviews, this moderate quantitative score showed a particular comprehension pattern rather than just being a statistical average. According to the qualitative data, this “moderate awareness” was defined by limited knowledge of the service's professional standards and only superficial familiarity with its existence. Participants indicated that they were aware that such a service existed, but they had misconceptions about its operational requirements.

The qualification requirements for nurses providing the service were a prime illustration of this disparity. “I think there should be no special requirements for home-based care; as long as you have a nursing license, it should be sufficient,” said a family caregiver (ID: B11, Family caregiver).

This quotation highlights a basic misconception: the idea that a general nursing license equates to the specialized skills needed to provide independent, home-based care. In actuality, “Internet + Nursing Service” requires providers to be licensed specialized nurses with further education. No participant correctly described the specialized qualification requirements, and these mistakes were common throughout the interviews.

Interpretive integration: The numeric “moderate perception” score is thus reframed as an “awareness–sophistication gap.” The public may not completely understand the specialized nature of the service, which could limit demand and institutional prioritization. This disparity between public comprehension and service complexity is a significant obstacle that the quantitative score does capture.

### Integrated theme 2: positive attitudes as conditional acceptance

Quantitative finding: Specialist nurses had an overall good attitude score of 3.90 ± 0.69. Female nurses and those employed in higher-level hospitals had significantly more favorable attitudes, according to univariate analysis (*p* < 0.05, Table 4).

**Table 4:** Specialist nurses' attitude to “Internet+Nursing Service” by different demographic characteristics (*n* = 1,200).

Characteristic	Results, *n* (%)	M ±SD	*t* or *F* test	*p*
Gender			−2.42	0.016
Man	62 (5.2)	3.70 ± 0.70		
Woman	1,138 (94.8)	3.92 ± 0.68		
Age (years)			1.23	0.183
20–29	183 (15.6)	3.84 ± 0.68		
30–39	703 (58.6)	3.89 ± 0.70		
40–49	276 (23.0)	3.99 ± 0.66		
50–59	38 (3.2)	3.95 ± 0.61		
Working experience			1.30	0.127
≤ 4	51 (4.3)	3.68 ± 0.62		
5–10	241 (20.1)	3.85 ± 0.74		
10–20	658 (54.8)	3.90 ± 0.67		
>20	250 (20.8)	4.03 ± 0.65		
Professional title			0.82	0.758
Nurse	41 (3.4)	3.84 ± 0.66		
Senior nurse	335 (27.9)	3.87 ± 0.71		
Supervisor nurse	722 (60.2)	3.90 ± 0.68		
Co-chief nurse	97 (8.1)	4.08 ± 0.65		
Chief nurse	5 (0.4)	3.54 ± 0.70		
Job position			1.30	0.122
Nurse	988 (82.3)	3.88 ± 0.68		
Head nurse	211 (17.6)	4.03 ± 0.69		
Director of nursing	1 (0.1)	3.52		
Educational level			1.33	0.104
Junior college or lower	72 (6.0)	4.00 ± 0.61		
Bachelor's degree	1,103 (91.9)	3.90 ± 0.69		
Master's degree or higher	25 (2.1)	3.94 ± 0.73		
Hospital type			−1.75	0.080
General hospital	1,058 (88.2)	3.89 ± 0.68		
Other specialist hospitals	142 (11.8)	4.00 ± 0.74		
Hospital level			1.78	0.005
Tertiary hospital	964 (80.3)	4.02 ± 0.54		
Secondary hospital	231 (19.3)	3.94 ± 0.71		
Primary hospital	5 (0.4)	3.90 ± 0.68		
Hospital nature			−0.83	0.408
Public hospital	1,177 (98.1)	3.90 ± 0.69		
private hospital	23 (1.9)	4.02 ± 0.63		
Authorized strength			1.83	0.068
Yes	323 (26.9)	3.96 ± 0.65		
No	877 (73.1)	3.88 ± 0.7		
The institution carrying out “Internet + Nursing Service”			2.24	0.025
Yes	482 (40.2)	3.96 ± 0.67		
No	718 (59.8)	3.87 ± 0.69		
Specialist nurse participated in”Internet+Nursing Service”			1.03	0.305
Yes	121 (10.1)	3.97 ± 0.74		
No	1,079 (89.9)	3.90 ± 0.68		

M, $\frac{1}{4}$ mean; SD, $\frac{1}{4}$ standard deviation.

Qualitative elaboration: Despite being sincere, this optimistic outlook was discovered to be conditional rather than absolute. Positivity coexisted with two key areas of concern, creating a complex pattern of “conditional acceptance,” according to a thematic analysis of patient and family interviews.

Sub-theme 2b: Convenience is the main source of happiness. The logistical advantages of the service were universally praised by participants. “*We no longer need to go to the hospital in person and wait in line; this saves us a lot of time and energy*,” one patient said, expressing relief from hospital-related obligations (ID: B8, Patient).

This sentiment was shared by a family caregiver who emphasized the importance of home-based care: “Internet + Nursing Services” is a really worthwhile project. “*This service allows us to care for the older adults at home without having to go*” (ID: B9, Family caregiver).

Sub-theme 2b: Cost and safety are important acceptance moderators. Convenience was welcomed, but this joy was tempered by real concerns that, if left unchecked, would reduce service uptake.

The lack of emergency medical facilities in homes was one of the main safety issues. “*Receiving treatment at home is not the same as in the hospital; without resuscitation equipment, it could be dangerous if something happens during the process*,” a patient succinctly explained this risk calculation (ID: B2, Patient).

The second main issue was economic barriers. One common issue was the perceived high cost in comparison to household income. “*The service is good, but the price… we have to think carefully every time we place an order*,” said a family member who specifically connected this issue to a possible legislative solution. “*We hope the medical insurance will cover it*” (ID: B4, Family caregiver).

Interpretive Integration: The qualitative results clarify why, despite being favorable, the quantitative attitude score does not correspond to widespread acceptance. Although the positive sentiments of specialist nurses (3.90 ± 0.69) may indicate their professional support of the service model, patients' and families' worries about the cost and safety are real-world modifiers that probably affect actual service utilization. Even nurses with favorable attitudes may detect limited demand or meet patient reluctance because of this conditional acceptance—positivity depending on safety assurances and pricing. Additionally, as such implementation would inevitably include safety procedures and pricing structures, the qualitative concerns clearly contextualize the quantitative finding that “institutional implementation” was a major predictor of nurses' impressions (Table 1).

### Integrated theme 3: structural and systemic challenges as barriers to scale-up

Quantitative finding: Together with employment position and educational level, the multiple linear regression analysis showed that institutional implementation (β = 0.35, *p* < 0.001) and individual engagement (β = 0.10, *p* = 0.003) were significant predictors of specialist nurses' perception scores (Table 5). This suggests that in addition to personal traits, nurses' perceptions of the service are influenced by whether their employer actively promotes it and whether they have personally contributed to its provision.

**Table 5:** Multiple linear regression analysis of factors associated with specialist nurses' perception scores (*n* = 1,200).

Variable	B (unstandardized)	SE	β (standardized)	*t*	*p*	95% CI for B
(Constant)	4.81	0.17	–	28.03	< 0.001	[4.48, 5.15]
Job position	0.13	0.04	0.08	3.03	0.003	[0.05, 0.22]
Educational level	−0.12	0.06	−0.05	−1.98	0.048	[−0.24, −0.001]
Institutional implementation	−0.32	0.04	−0.25	−8.63	< 0.001	[−0.39, −0.25]
Indiviual participation	−0.48	0.06	−0.23	−8	< 0.001	[−0.60, −0.36]

CI, confidence interval; SE, standard error. The final model was significant (*F* = 57.76, *p* < 0.001) and explained 15.9% of the variance (adjusted *R*^2^ = 0.159). VIF values for all predictors were < 2.0, indicating no multicollinearity.

Qualitative elaboration: Rich contextual information from patient and family interviews explained why “institutional implementation” emerged as such a powerful predictor. Participants discussed systemic issues that become obstacles to service acceptability when institutions fail to address them.

Sub-theme 3a: Incomplete fee and reimbursement systems: One frequently mentioned obstacle was the lack of a clear and reasonably priced payment plan. “*We hope the costs will be covered by medical insurance*,” one participant said, expressing anxiety regarding costs and a desire for structural solutions. “*Otherwise, it's a luxury that our family can only sometimes afford*” (ID: B4, Family caregiver).

Sub-theme 3b: Inadequate safety procedures: A perceived lack of institutional safeguards was associated with safety concerns. This discrepancy between home-based care and institutional norms (seen in hospitals) is reflected in one patient's statement: “*Safety is our primary concern*. *Who is in charge if something goes wrong at home? There are emergency teams and protocols in the hospital*”(ID: B2, Patient).

Sub-theme 3c: Barriers to digital literacy: In terms of service accessibility, a third systemic issue surfaced. Older adult patients have trouble using the digital platform needed to order services, according to several participants: “*My mother is 82. She is completely unable to utilize the app. Every time, I have to assist her. How would some of her friends who don't have kids nearby even use this service?*” (ID: B7, Family caregiver).

Interpretive Integration: The quantitative regression results' explanatory mechanism is derived from the qualitative findings. The strong correlation between “institutional implementation” and nurses' perceptions can be explained as follows: nurses directly suffer the consequences—patient reluctance, service refusal, or operational confusion—when institutions fail to establish clear safety protocols, transparent fee structures, or easily accessible digital platforms. Nurses' opinions of the service's viability and worth are subsequently influenced by these encounters. On the other hand, nurses who work in organizations with strong implementation (such as user support, insurance integration, and explicit guidelines) probably form more accurate and positive opinions. This integration shows that the quantitative predictor, “institutional implementation,” is not just a statistical artifact but rather a collection of tangible structural elements, such as cost, accessibility, and safety, that significantly influence provider attitudes and service uptake.

## Discussion

### Synthesis of mixed-methods insights

This convergent mixed-methods study combined qualitative interview data from 12 patients and family caregivers in Yunnan Province, China, with quantitative survey data from 1,200 specialized nurses. Three primary themes emerged from the integrated analysis: (1) Moderate awareness with a nuanced understanding gap; (2) Positive attitudes as conditional acceptance; and (3) Structural and systemic challenges as barriers to scale-up. In the sections that follow, we revisit the research questions from the introduction, analyze each result in light of the body of existing literature, and draw conclusions.

### Key findings interpretation: addressing the research questions

RQ1: How do specialist nurses in Yunnan Province perceive and feel about Internet + Nursing Services?

Our quantitative results revealed a mean attitude score of 3.90 ± 0.69 (positive) and a mean perception score of 3.31 ± 0.64 (moderate and slightly positive). These findings support H1 to some extent. In line with earlier research from coastal Chinese cities, the perception score is lower than the attitude score (18, 19). But the qualitative component showed that this “moderate perception” represented an awareness–sophistication gap rather than just a neutral midpoint (Theme 1): Participants showed a cursory awareness of the service's existence, but they had basic misconceptions about its specific requirements (such as a general nursing license suffices). By demonstrating that assessing awareness on a Likert scale without probing depth of understanding may overestimate true knowledge, this qualitative elaboration builds on earlier quantitative research. From a global standpoint, this disparity might be especially noticeable in environments where Internet + Nursing Service is relatively new, public health awareness of advanced nursing roles is low, and regulatory frameworks are still developing (20).

RQ2: What variables are connected to the perception and attitude scores of nurses?

Job position, educational level, institutional implementation, and individual participation were found to be significant predictors (all *p* < 0.05) using the multiple linear regression (Table 5). Partially supporting H2, institutional implementation had the strongest association (β = 0.35, *p* < 0.001). Notably, although univariate analysis revealed that female nurses and those working in higher-level hospitals had more positive attitudes, these factors did not hold significance in the adjusted model, indicating that institutional factors have a greater influence on perceptions than demographic traits.

The strongest predictor, institutional implementation, was explained mechanistically by the qualitative data. Concerns over safety procedures (e.g., “without resuscitation equipment, it could be dangerous”), financial obstacles (e.g., “it's a luxury we can only afford occasionally”), and digital accessibility (e.g., “my mother is 82—she can't use the app”) were expressed by patients and their families. These issues exactly correspond to the areas that well-executed institutional regulations would cover (Table 6): multichannel access, transparent fee structures, and unambiguous emergency response procedures. Because they see fewer patient refusals and operational challenges, nurses who work in institutions with strong implementation are likely to have more accurate and positive opinions. This result is consistent with global research on telehealth adoption, which has demonstrated that provider acceptance is more strongly predicted by organizational support (such as training, reimbursement systems, and safety procedures) than by personal traits (20).

**Table 6:** The factors that specialized nurses considered to be obstacles to the promotion and development of “Internet+Nursing Service” (*n* = 1,200).

Variable	*n* (%)
Relevant laws are not perfected and policy support is insufficient	987 (82.3)
Lack of uniform industry standards, fee schedules, performance evaluation mechanisms and a sound medical insurance system	814 (67.8)
Nursing human resources are in short supply and multi-practice nursing has not yet been fully opened up	1,130 (94.1)
Multiple risks associated with home-based services (e.g., medical risks, personal accident risks, medical disputes, etc.)	1,005 (83.8)
Low motivation of caregivers to participate in “Internet+Care” home services	859 (71.6)
Patients' traditional attitudes (to hospitals) are difficult to change for a while	566 (47.2)
Most of the service users are elderly people, who are less capable of accepting new things and do not apply on-line	912 (76.0)
other	116 (9.7)

RQ3: How do the experiences of patients and their families clarify, interpret, or add to the quantitative results?

Three distinct levels of elaboration emerged from the qualitative themes. First, the date reinterpreted the moderate perception score in a particular qualitative pattern that would be undetectable in a strictly quantitative study: the awareness–sophistication gap. Second, the data demonstrated that positive attitudes are not absolute but rather conditional (Theme 2). Convenience was highly valued by patients and their families, but safety concerns, financial worries, and unclear responsibilities limited their acceptance. Even nurses with favorable attitudes may perceive low demand or experience patient resistance, which can be explained by this conditional acceptance. Third, the qualitative data revealed the specific structural elements underlying the statistical relationship with institutional implementation. Overall, these findings indicate that the primary constraints to service scale-up in this regional context are structural and informational rather than attitudinal.

### Comparing the theoretical framework and current literature

The combined results of this study complement and build upon earlier research. This study also discovered favorable sentiments among Chinese nurses, which is in line with Li et al. (18) and Ma et al. (19). However, the awareness–sophistication gap identified by this study has not been documented before, probably because earlier research did not use mixed approaches to examine the depth of comprehension. International research on home-based telehealth in the United States and Australia has revealed similar safety issues and limitations in digital literacy, indicating that these issues may apply to any internet-based care models. However, the study discovers that these issues specifically affect institutional implementation (β = 0.35) and provides a more accurate target for intervention than was previously possible.

**Figure 1:**
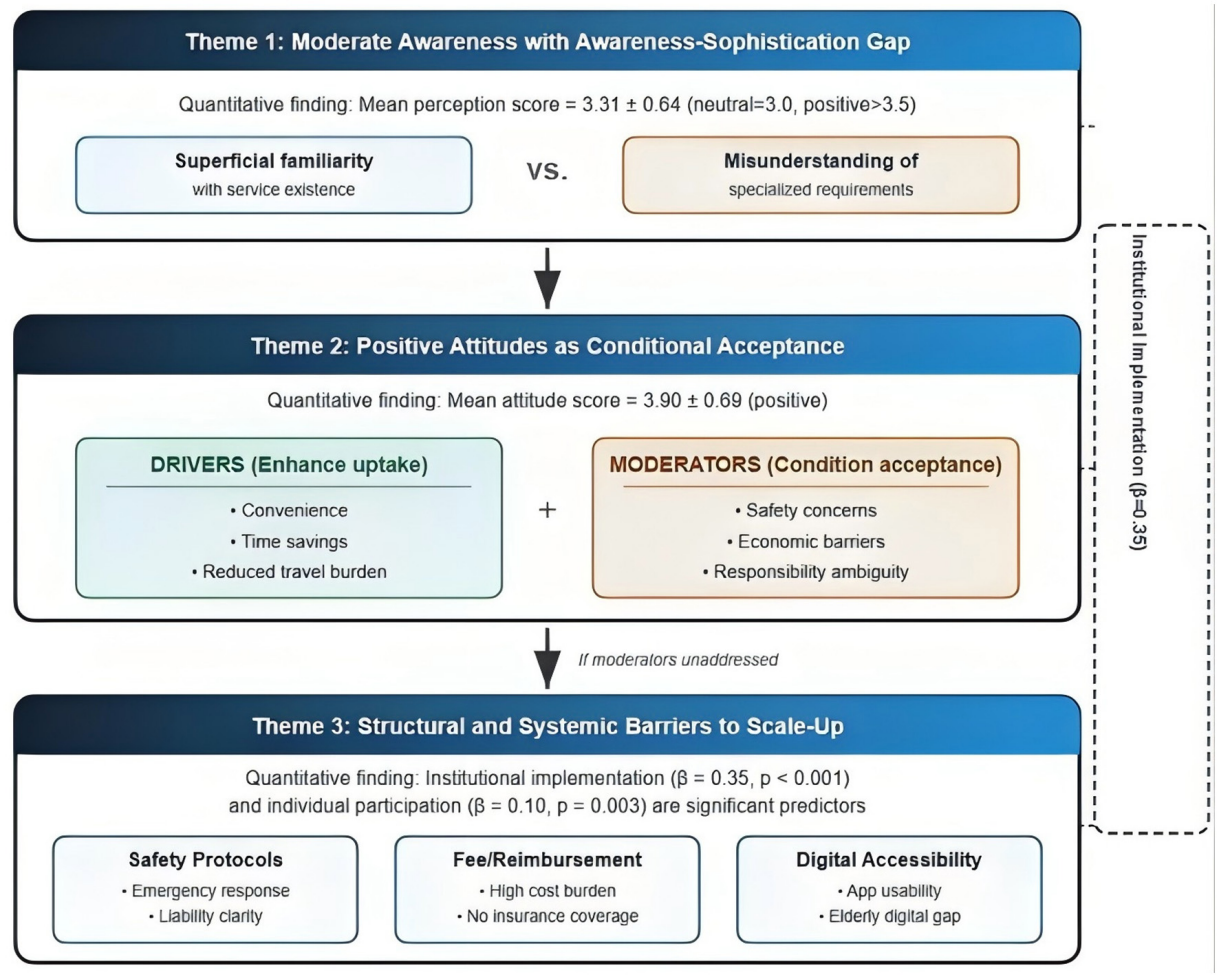
Conceptual framework of intergrated findinds.

In theory, a conditional acceptance model is proposed by the integrated framework (Figure 1). According to this paradigm, service uptake is influenced by perceived safety, cost, and accessibility rather than being only driven by positive attitudes. By including structural impediments as important variables that are not captured by perceived usefulness or ease of use alone, this depends on the Technology Acceptance Model (TAM) (21). Additionally, this study model emphasizes the significance of public knowledge (such as awareness–sophistication gap) as a distal determinant. This is especially important in situations where the service model is new, and the general public is not fully aware of the specialized nursing duties.

## Recommendations

Close the awareness–sophistication gap: Rather than just promoting service availability, public awareness campaigns should explain the specialized training of Internet + Nursing Service providers (such as “certified specialist nurses only”). Posters in hospital discharge units, patient testimonials highlighting qualifying requirements, and brief instructional movies are a few examples of this.

Boost institutional implementation: Hospital managers should prioritize explicit safety procedures for home-based care (such as emergency response plans, liability agreements, and real-time monitoring through video conferences). Insurance reimbursement options should be tested in pilot projects, as cost was a persistent obstacle. To address hurdles to digital literacy, multi-channel service access (such as telephone booking and community health worker support) should complement digital platforms.

Pre-licensure and continuing nursing education curricula should include Internet + Nursing Service content, with emphasis on communication tactics to convey specialist qualifications to patients and families, along with technical skills for home-based care.

Our conclusions are based on associative evidence and a cross-sectional approach. To assess the efficacy of the suggested strategies—for example, does a focused educational effort close the awareness-sophistication gap?—further longitudinal or interventional research is required. Is service uptake increased by pilot insurance coverage? Causal evidence for the role of institutional implementation would come from a cluster-randomized study that varied implementation intensity among hospitals.

## Transferability of findings

The study was carried out in Yunnan Province, China, which is distinguished by its hilly topography, ethnic diversity, and lower levels of economic development than China's coastal regions. The results may be applicable to other middle-income, geographically scattered, or ethnically varied areas where Internet + Nursing Service is being implemented. Examples include underserved areas in high-income countries (such as remote Aboriginal communities in Australia and Appalachia in the United States), rural parts of Latin America (such as Andean regions), and portions of Southeast Asia (such as northern Vietnam and Laos).

Transferability is not always possible. In terms of: (a) digital infrastructure and broadband access; (b) health insurance systems (particularly coverage for home-based care); (c) cultural attitudes toward home care vs. institutional care; (d) regulatory frameworks for nursing practice and liability; and (e) the maturity of the Internet + Nursing Service model (early implementation vs. established service), researchers and policymakers should evaluate contextual similarities and differences. Different economic development levels, the availability of specialized nurses, and local governmental support are some of the factors that could restrict transferability to other Chinese provinces.

## Strengths and limitations

**Figure 2:**
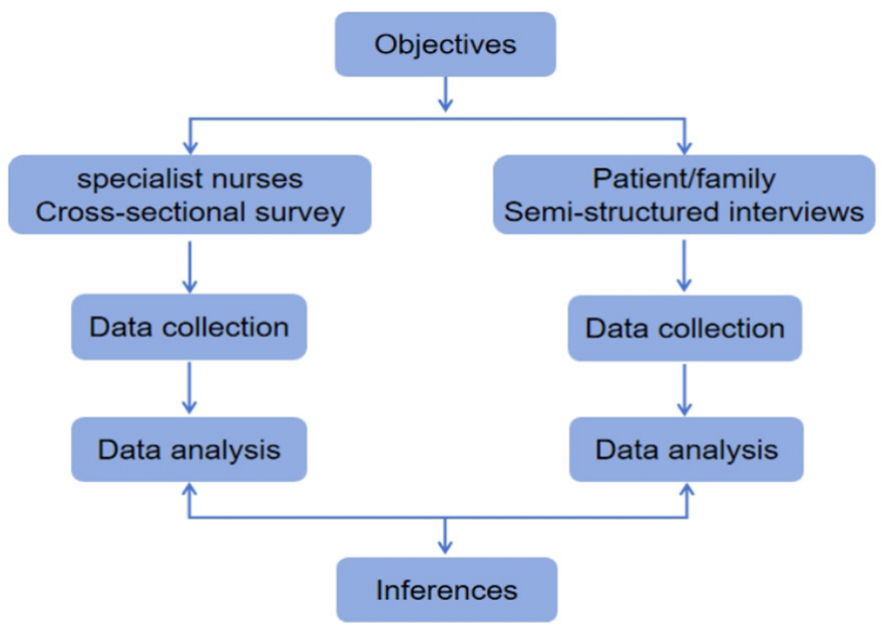
Srtudy structure.

Strengths: A more thorough insight is provided by this study's mixed-methods design, which combines provider viewpoints (*n* = 1,200) with receiver experiences (*n* = 12). Qualitative themes were able to explain quantitative patterns through the convergent design with equal priority (weaving technique). The conceptual framework (Figure 2) and unified display (Table 1) improve transparency.

Theoretical limitations: The conditional acceptance model remains exploratory and is based on a single region (Yunnan Province). Cross-sectional data cannot support causal inferences, and prospective testing in diverse settings is needed to validate the proposed relationships.

Methodological limitations: First, selection bias may have been introduced by the convenience sampling strategy used for nurse surveys; there may be systematic disparities between participants and non-participants (such as lack of interest in Internet + Nursing Service or limited digital literacy). Second, social desirability bias affects self-reported data (including both survey and interview); this study attempted to reduce this bias by maintaining anonymity and using neutral probing. Third, the study was unable to follow non-respondents or differentiate between early and late respondents to evaluate non-response or early–late bias because the survey was distributed anonymously and electronically. Systematic disparities between responders and non-respondents cannot be completely ruled out, despite the high response rate of 92.9%. Fourth, because the study was limited to a single Chinese province, its conclusions may not apply to other areas with distinct legal frameworks, cultural norms, or healthcare financing systems.

## Conclusion

The study was carried out in Yunnan Province, China, a hilly, multiethnic, and economically disadvantaged province that lags behind coastal areas in terms of institutional capability and digital infrastructure for “Internet + Nursing Service” (Internet + Nursing Service). There are three key conclusions.

First, specialized nurses had a moderate assessment (3.31 ± 0.64) but good sentiments (3.90 ± 0.69). According to qualitative data, this moderate score concealed an awareness-sophistication gap, wherein a lack of knowledge of specific certification needs coexisted with superficial acquaintance.

Second, although patients and their families enjoyed convenience, they also voiced serious safety and financial concerns, suggesting that favorable opinions of Internet + Nursing Service are conditional rather than unconditional.

Third, nurses' perception scores were best predicted by institutional implementation (β = 0.35, *p* < 0.001). Concrete structural barriers, such as insufficient safety procedures, incomplete fee/reimbursement systems, and difficulties with digital literacy, were found in qualitative data to explain this association.

These conclusions are unique to Yunnan Province. A thorough evaluation of the local infrastructure, economy, and regulations is necessary for transferability. Studies that replicate in different contexts are required. Key leverage points for hospital administrators and policymakers in comparable resource-constrained environments include: (1) focused awareness campaigns outlining service specialization; (2) systemic reforms to set safety procedures and insurance coverage; and (3) multi-channel access (such as telephone booking) to overcome digital obstacles.

## Data Availability

The original contributions presented in the study are included in the article/supplementary material, further inquiries can be directed to the corresponding author.
